# A versatile experimental system for tracking ultrafast chemical reactions with X-ray free-electron lasers

**DOI:** 10.1063/1.5111795

**Published:** 2019-09-12

**Authors:** Tetsuo Katayama, Shunsuke Nozawa, Yasufumi Umena, SungHee Lee, Tadashi Togashi, Shigeki Owada, Makina Yabashi

**Affiliations:** 1 Japan Synchrotron Radiation Research Institute, 1-1-1 Kouto, Sayo, Hyogo 679-5198, Japan; 2 RIKEN SPring-8 Center, 1-1-1 Kouto, Sayo, Hyogo 679-5148, Japan; 3 Institute of Materials Structure Science, High Energy Accelerator Research Organization (KEK), 1-1 Oho, Tsukuba, Ibaraki 305-0801, Japan; 4 Department of Materials Structure Science, School of High Energy Accelerator Science, The Graduate University for Advanced Studies, 1-1 Oho, Tsukuba, Ibaraki 305-0801, Japan; 5 Research Institute for Interdisciplinary Science and Graduate School of Natural Science and Technology, Okayama University, 3-1-1 Tshushima Naka, Okayama 700-8530, Japan; 6 Department of Chemistry and Chemical Institute for Functional Materials, Pusan National University, Busan 609-735, South Korea

## Abstract

An experimental system, SPINETT (SACLA Pump-probe INstrumEnt for Tracking Transient dynamics), dedicated for ultrafast pump-probe experiments using X-ray free-electron lasers has been developed. SPINETT consists of a chamber operated under 1 atm helium pressure, two Von Hamos spectrometers, and a large two-dimensional detector having a short work distance. This platform covers complementary X-ray techniques; one can perform time-resolved X-ray absorption spectroscopy, time-resolved X-ray emission spectroscopy, and time-resolved X-ray diffuse scattering. Two types of liquid injectors have been prepared for low-viscosity chemical solutions and for protein microcrystals embedded in a matrix. We performed a test experiment at SPring-8 Angstrom Compact free-electron LAser and demonstrated the capability of SPINETT to obtain the local electronic structure and geometrical information simultaneously.

## I. INTRODUCTION

X-ray free-electron lasers (XFELs),^1–5^ generating unprecedentedly brilliant coherent X-ray pulses with an ultrashort duration (∼10 fs), have boosted femtochemistry research activities. Beyond the optical-domain observables, XFELs offer exciting opportunities to observe structural and local electronic changes of matter in ultrafast pump-probe experiments. To date, time-resolved X-ray spectroscopic or scattering measurements using XFELs have revealed various fundamental phenomena such as a covalent bond formation,^6,7^ photocarrier generation, and trapping inside semiconductor nanoparticles,^8–10^ a ligand dissociation^11^ or exchange^12^ of transition metal complexes, and coherent molecular vibrations associated with the nuclear wavepacket dynamics.^13–16^

A combination of complementary X-ray techniques in ultrafast experiments is useful for disentangling the complexity of excited-state processes and allows a comprehensive understanding of the nonequilibrium reaction dynamics. For example, time-resolved X-ray absorption spectroscopy (TR-XAS) can probe both the unoccupied electronic states and local nuclear structure around an absorbing atom, while time-resolved X-ray emission spectroscopy (TR-XES) provides information about the occupied density of states projected on the absorbing atom. Time-resolved X-ray diffuse scattering (TR-XDS) is an important tool to obtain structural insights from an interference pattern of elastically scattered photons. The usefulness of these combinations is well illustrated in the work by Canton and co-workers,^17^ who exploited TR-XES and TR-XDS to investigate a bimetallic donor–acceptor complex and determined the time scales of the intramolecular charge transfer and the subsequent structural change.

At BL3 of SPring-8 Angstrom Compact free-electron LAser (SACLA),^1^ we have developed advanced X-ray optics and beamline components for accommodating these applications. The most widely used system is an arrival-timing monitor based on a beam-splitting scheme.^18,19^ With this diagnostics, one is able to measure shot-to-shot timing fluctuations between the optical pump and X-ray probe pulses during the experiments, to compensate for them with postprocess analysis, and to improve the time resolution to the ∼20 fs level.^20^ Later, we also implemented a double channel-cut crystal monochromator (DCCM) and compound refractive lenses (CRLs),^21^ which are compatible with the arrival-timing diagnostics and facilitate to tailor the XFEL beam conditions, i.e., bandwidth and beam size. These fundamental beamline components are working reliably.

For an efficient operation of ultrafast XFEL experiments at SACLA, we have also developed a robust measurement system, SPINETT (SACLA Pump-probe INstrumEnt for Tracking Transient dynamics), covering complementary X-ray techniques. This experimental platform allows one to carry out TR-XAS, TR-XES, and TR-XDS measurements of various molecular and protein samples. In this paper, we describe the design concept and functionality of SPINETT. A test experiment was performed to demonstrate instrumental capabilities. In the conclusion, we briefly discuss future perspectives.

## II. DESIGN

### A. Overview

**FIG. 1.**
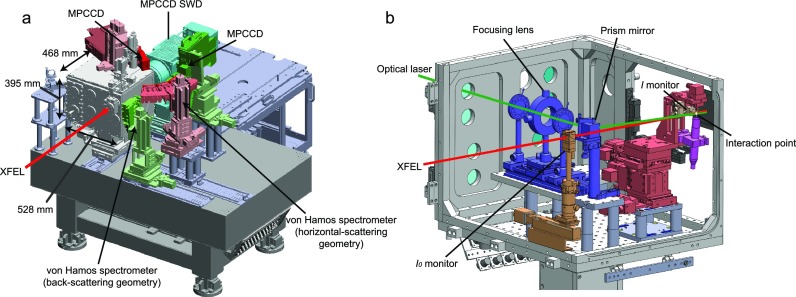
Schematic layout of SPINETT. (a) The whole view. (b) Components inside the He chamber. The XFEL and optical pulse trajectories are drawn as red and green lines, respectively.

Figure 1(a) shows a schematic layout of SPINETT, consisting of a chamber with a size of 528 × 468 × 395 mm^3^, two Von Hamos spectrometers, and a multiport charge-coupled device (MPCCD) detector^22^ with a short working distance (SWD). The chamber is separated from the beamline by a polyimide film and can be filled with helium gas at a pressure of 1 atm to suppress the scattering and absorption of the incident X-ray beam by air. Statistics of SPINETT are summarized in Table I.

**TABLE I.** Statistics of SPINETT.

Description	Parameter
X-ray energy	4 keV–15 keV
XFEL beam size at the interaction point	>1 *μ*m^a^ or >1.5 *μ*m^b^
XFEL intensity per pulse (monochromatic beam)	∼10 *μ*J at 10 keV^c^
XFEL intensity per pulse (pink beam)	∼600 *μ*J at 10 keV^d^
XFEL pulse duration	<10 fs
Optical wavelength	200 nm–2000 nm
Optical pulse duration (800 nm, full width at half-maximum; FWHM)	30 fs
Achievable time resolution (FWHM)	70 fs^e^
Bragg angle range of Von Hamos spectrometers	60°–75°
Maximum momentum transfer (**Q**) available for TR-XDS (10 keV, 15 keV)	3.88 Å^−1^, 5.82 Å^−1^

^a.^ This corresponds to the focal beam size obtained with the Kirkpatrick-Baez (KB) mirrors.^32^

^b^ This corresponds to the focal beam size obtained with the CRLs.^21^

^c^ XFEL intensity after the four-bounced Si(111) reflections by the double channel-cut crystal monochromator^21^ implemented at SACLA BL3.

^d^ This is the typical case of SACLA BL3.

^e^ The time resolution depends on the optical pulse duration, the jet thickness, and other experimental conditions. This value was evaluated in Ref. 16.

### B. Optical laser path

Optical pulses are introduced into the chamber through a 1-mm-thick quartz window attached to a sidewall and are delivered to the interaction point with a collinear geometry via a prism mirror. The incident angle difference between optical pump and X-ray probe pulses is ∼4°. The optical beam is focused by a lens located upstream of the prism mirror. The lens position is adjustable to control the optical beam size at the interaction point.

### C. Injectors

**FIG. 2.**
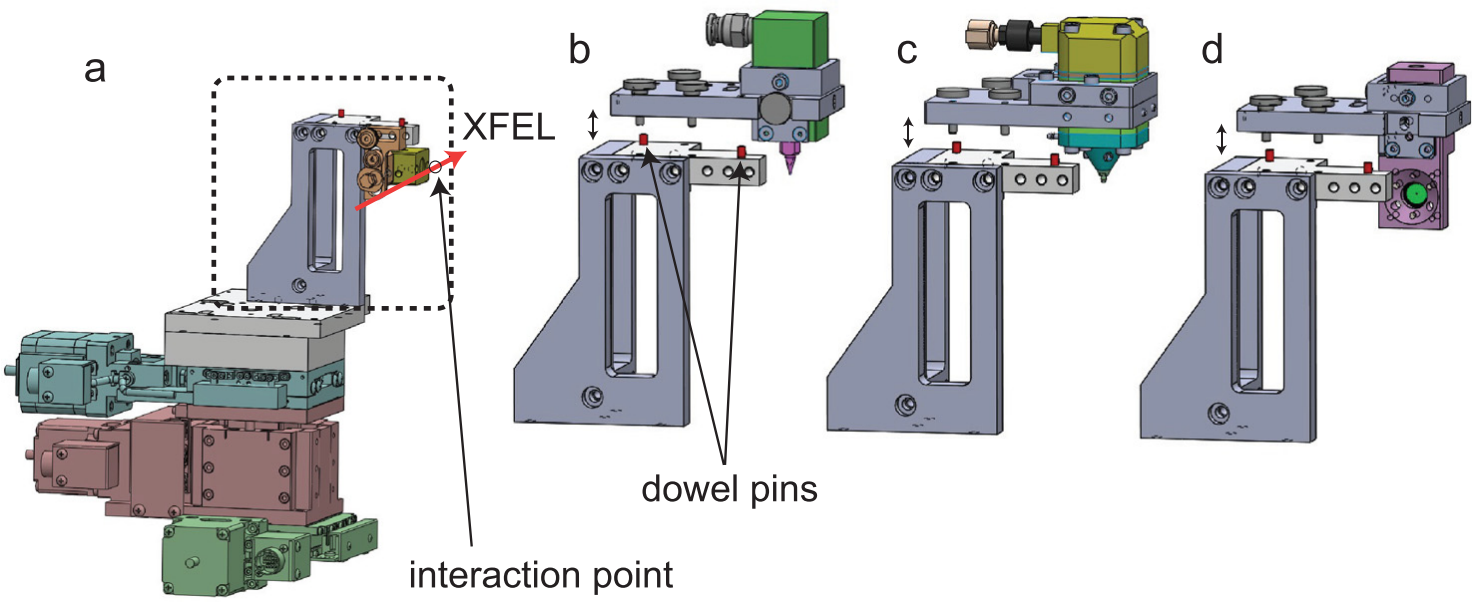
Drawings of (a) the manipulator, (b) and (c) the injectors, and (d) the pinhole. The injector (b) is dedicated for chemical solutions, while the injector (c) is designed for protein microcrystals. The typical inner diameters of nozzles are 30, 50, 100, 150, 200, 300, and 500 *μ*m for (b) and 50, 75, 100, 125, 150, and 200 *μ*m for (c). The injectors and pinhole are replaceable.

Figure 2(a) shows a motorized manipulator used to mount two types of liquid-jet injectors and a pinhole. One of the injectors is a commercial product [Musashi Engineering, Super-fine nozzle FN; Fig. 2(b)] for circulating a low-viscosity chemical solution. The other is a syringe injector [Fig. 2(c)] for extruding protein microcrystals embedded in a matrix carrier such as grease^23^ or cellulose.^24^ To rapidly achieve a spatial overlap between XFEL and optical laser pulses, we replace these injectors with a tungsten pinhole having a diameter of 50 *μ*m [Fig. 2(d)], move its center hole to the XFEL beam, and align the optical beam with a motorized steering mirror. The surface of the pinhole is coated with phosphor (Gd_2_O_2_S:Tb, P43) powder via a heterogeneous precipitation method and emits visible light when XFEL or optical laser pulses are irradiated. These components are assembled on the manipulator through dowel pins to ensure a high positional (∼50 *μ*m) repeatability.

### D. Intensity monitors

**FIG. 3.**
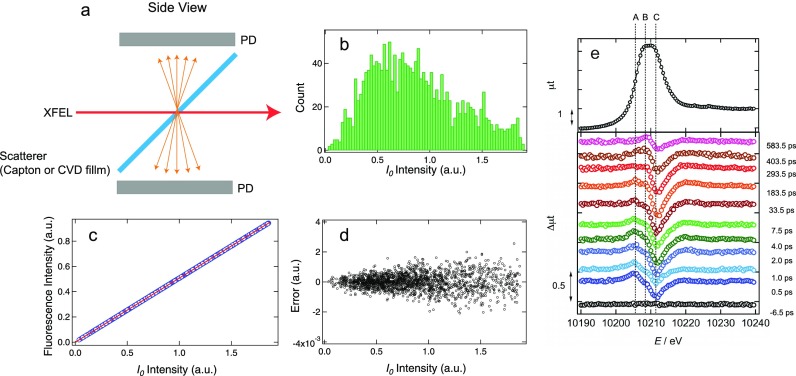
X-ray intensity monitor for TR-XAS. (a) Schematics of the *I*_0_ intensity monitor. (b) Monochromatic XFEL intensity fluctuation at 8.985 keV obtained using ∼1800 shots. (c) Correlation of the fluorescence intensity with respect to incoming monochromatic intensity for the same shots with (b). The used sample is a [Cu^I^(dmphen)_2_]^+^ (dmphen = 2,9-dimethyl-1,10-phenanthroline) dissolved in acetonitrile with a concentration of 100 mM. The solution was flowed as a liquid jet through the injector with an inner diameter of 50 *μ*m. The blue circles and red line correspond to the experimental data and the linear fitting, respectively. (d) The residual error after the linear fitting of (c). (e) W L_III_ TR-XAS spectra. (Reproduced with permission from Uemura *et al.* “Dynamics of photoelectrons and structural changes of tungsten trioxide observed by femtosecond transient XAFS,” Angew. Chem., Int. Ed. **55,** 1364–1367 (2016). Copyright 2016 Wiley-VCH Verlag GmbH & Co. KGaA.) The sample was the water suspension of WO_3_ nanoparticles with a concentration of 4 mM, which was flowed through the injector with a diameter of 500 *μ*m.

TR-XAS can be measured with a total fluorescence detection method using monochromatic XFELs, whose intensity fluctuates stochastically owing to the self-amplified spontaneous emission (SASE) process. Consequently, the data quality strongly depends on the precision with which the incoming and fluorescence X-ray intensities (*I*_0_ and *I*) are measured. In order to achieve a high signal-to-noise (S/N) ratio in TR-XAS, *I*_0_ and *I* intensity monitors are installed inside the chamber. The *I*_0_ transmissive intensity monitor consists of an X-ray scatterer (a polyimide or CVD film) and two Si photodiodes (Hamamatsu, S3590–09) as shown in Fig. 3(a). These photodiodes produce charge pulses upon X-ray irradiation, which are processed by the charge-sensitive and shaping amplifiers. The output signals from the amplifiers are evaluated by a waveform fitting procedure and the *I*_0_ intensity can be measured with a relative accuracy of ∼10^−3^, as described in Ref. 25. The *I*_0_ intensity fluctuation can be defined as the standard deviation over the average *I*_0_ intensity ($\sigma_{I_{0}}/\bar{I_{0}}$), which was measured to be ∼50% at 8.985 keV [Fig. 3(b)]. Despite such large intensity fluctuations of monochromatic XFELs, the normalization can be achieved by using another Si photodiode to measure the fluorescence intensity, which is placed at ∼10 mm from the interaction point so as to cover a large solid angle (∼0.81 sr). Figure 3(c) shows shot-to-shot fluorescence intensity plotted against the *I*_0_ intensity, which corroborates an excellent linear correlation between them. This is also confirmed by the residual error after the linear fitting, as shown in Fig. 3(d). Figure 3(e) is reprinted from Ref. 9, showing W L_III_ TR-XAS of WO_3_ nanoparticles measured using SPINETT. The top shows the spectrum of WO_3_ in the ground state and the bottom shows the difference spectra of WO_3_ in the excited state.

### E. Von Hamos spectrometers

**FIG. 4.**
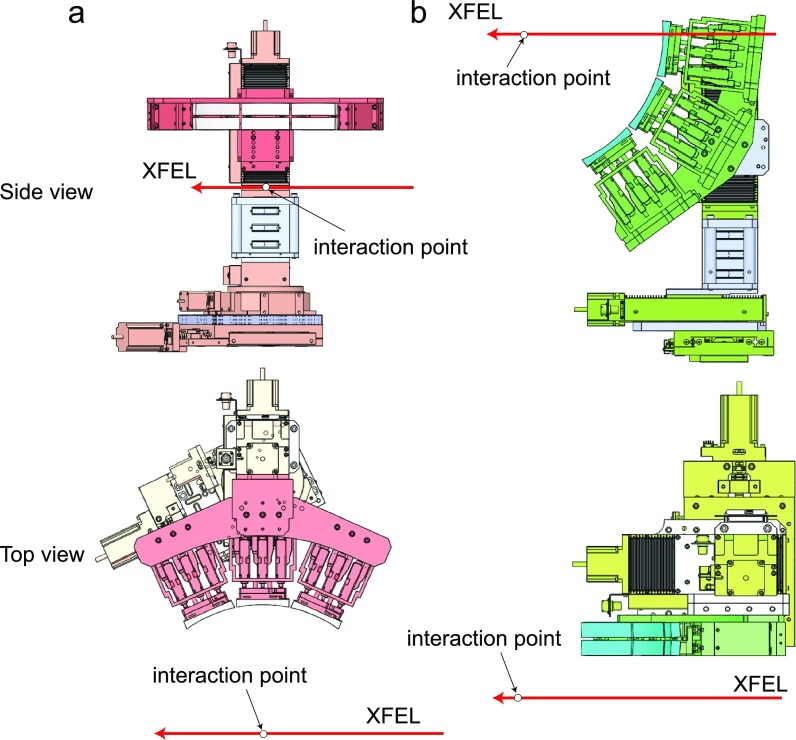
Drawings of two Von Hamos spectrometers. (a) Horizontal-scattering geometry. (b) Back-scattering geometry.

The two Von Hamos spectrometers consist of 6 cylindrically bent crystals (Fig. 4). The crystals are each 25 mm × 100 mm × 150 *μ*m (in the dispersive direction, the focusing direction, and the thickness, respectively), which is glued onto the cylindrical glass lens with a radius of curvature of 250 mm. The shape errors of the crystal surfaces were measured with an optical interferometer (Zygo Verifire^TM^ ATZ) and were kept within ∼0.4 *μ*m in root mean square over 90% of the surface area. The available crystals are Si(531) and Si(111). In the future, we plan to fabricate analyzer crystals with different Miller indices or materials, such as Si(220), Ge(111), and Ge(220). The Bragg angle range covered by these spectrometers is from 60° to 75°. One of the spectrometers is positioned at 90° with respect to the incident X-ray beam [horizontal-scattering geometry; Fig. 4(a)], while the other has a back-scattering geometry [Fig. 4(b)]. Owing to these two spectrometers, multiple signals can be measured simultaneously, for example, Kα and Kβ emissions of 3d transition metals. Two MPCCD detectors, which have an area of 25.6 mm × 51.2 mm with a pixel size of 50 *μ*m, are used for XES measurements. These detectors are positioned at the top and at the side of the interaction point, depending on the reflection geometry.

### F. MPCCD SWD detector

**FIG. 5.**
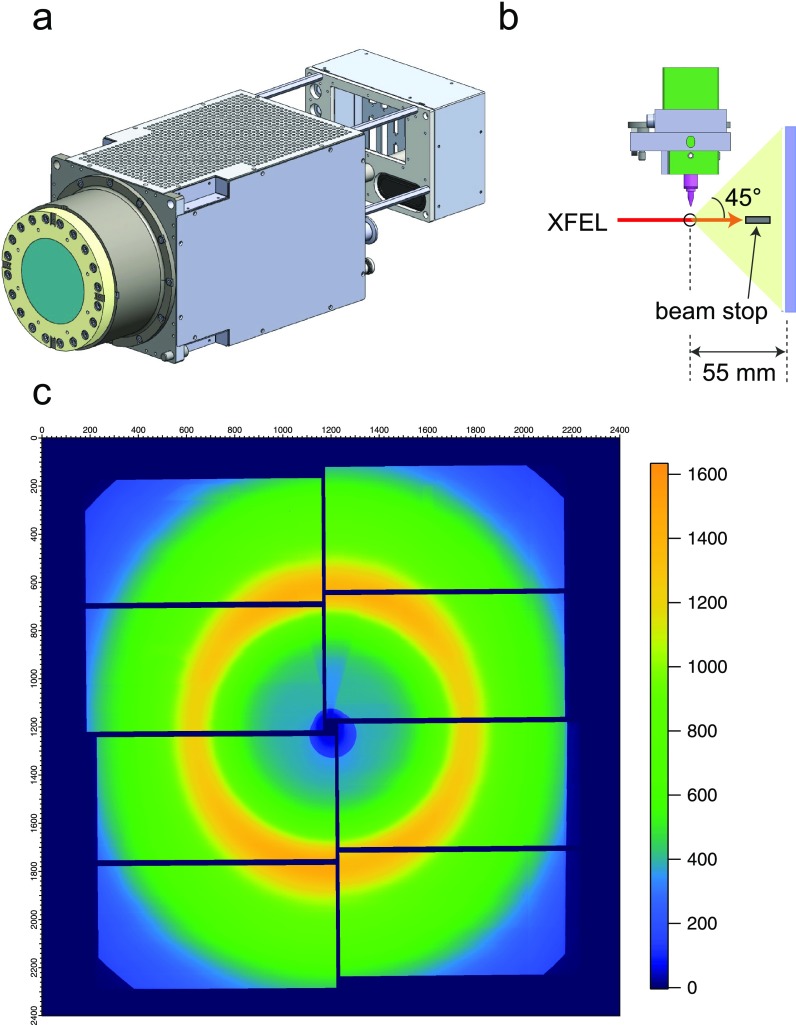
Instruments for TR-XDS. (a) Drawing of MPCCD with a short working distance. (b) Side view of the injector and MPCCD in the X-ray scattering or diffraction measurement. (c) The XDS image averaged over 10 000 shots. The XFEL energy was 9 keV and the camera distance was set at 55 mm.

The MPCCD SWD detector shown in Fig. 5(a) is composed of eight sensor modules and constitutes a square sensor area of 110 mm × 110 mm with a square aperture of 3 mm × 3 mm at the center. This detector is mounted downstream of the chamber and allows measuring XDS or X-ray diffraction (XRD) from the sample. The working distance between the sample and the sensor surface is adjustable in the range of 55 mm–1400 mm, while we usually set it at the minimum of 55 mm in order to reach a high scattering angle (2θ) of 45° [Fig. 5(b)]. In the experiment, we block the incident XFEL beam with a 3-mm-diameter beam stopper equipped inside the chamber to prevent damage to the detector. Figure 5(c) shows the averaged XDS image measured for the [Co^II^(bpy)_3_]^2+^ (bpy = bipyridine) complex dissolved in water with a concentration of 10 mM. The solution was flowed as a liquid jet through the injector [Fig. 2(b)] with an inner diameter of 100 *μ*m in a closed circulating system. An intense ring in Fig. 5(c) arises from the solvent scattering.

## III. PERFORMANCE TEST

The data quality of TR-XAS measured using SPINETT can be found in Refs. 8–11 and 16. In this work, we report a test experiment investigating the electronic ground structures of Fe atoms by XES. The samples were 50 mM [Fe^II^(phen)_3_]^2+^ (phen = phenanthroline) aqueous solution and photosystem II (PSII) microcrystals. The PSII preparation procedure is described elsewhere.^26–29^ A 100 *μ*l PSII suspension was mixed with a 3 ml grease matrix^23^ without postcrystallization treatment and size-screening. This resulted in a variation of the microcrystal size ranging from 10 *μ*m to 100 *μ*m. We employed injectors with inner diameters of 100 *μ*m for the [Fe^II^(phen)_3_]^2+^ solution and 150 *μ*m for the PSII microcrystals. In the XES measurement of PSII, XRD was collected simultaneously. Diffraction images were filtered by the program of Cheetah,^30,31^ which allows us to extract the events when the XFEL pulses are diffracted by the microcrystals.

The experiment was performed at BL2 of SACLA. We used the pink XFEL beam with an average pulse energy of 470 *μ*J and a central photon energy of 8 keV. At the interaction point, the beam was focused to a full-width at half-maximum (FWHM) spot size of 1.5 *μ*m × 1.5 *μ*m by Kirkpatrick-Baez mirrors.^32^ Fe Kβ spectra were recorded with the Si(531) reflection (Bragg angle = 73.14°) using the horizontal-scattering geometry, while Fe Kα spectra were measured with the Si(333) reflection (Bragg angle = 67.89°) using the back-scattering geometry. The elastic peaks of the monochromatic beam through the Si(111) monochromator gave 1.1 eV (1.0 eV) FWHMs for the Kβ (Kα) region. After deconvoluting the monochromator contribution, the energy resolutions were evaluated to be ∼0.5 eV for Kβ and ∼0.6 eV for Kα, which are mainly due to the geometrical contribution^33,34^ and the misalignment in overlapping analyzer crystals (∼0.3 eV). The geometrical contribution $\Delta E_{G}$ can be described as

$$\begin{array}{c} \Delta E_{G}=\Delta \theta \cdot \text{cot}(\theta_{B})\\ \Delta \theta \approx (s+d)/f, \end{array}$$ (1)

where s, d, f, and $\theta_{B}$ are the source size (1.5 *μ*m), the detector pixel size (50 *μ*m), the distance between the analyzer crystal and the detector, and the Bragg angle, respectively. The $\Delta E_{G}$ values are calculated to be ∼0.4 eV for Kβ and ∼0.5 eV for Kα.

**FIG. 6.**
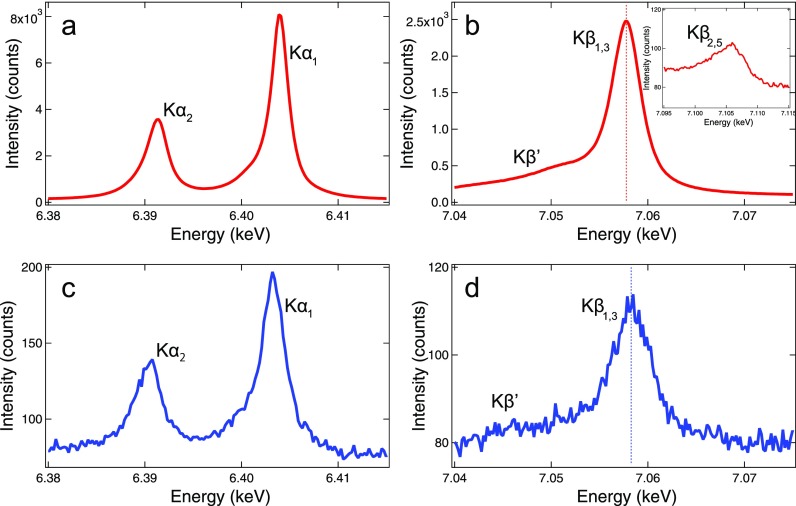
XES of (a) and (b) [Fe^II^(phen)_3_]^2+^ solution and (c) and (d) PSII microcrystals. The vertical axis indicates the total counts. The dotted lines in (b) and (d) correspond to the Kβ_1,3_ peak center. The shot number used in (a) and (b) was 90 000 (∼50 min at 30 Hz), while the program Cheetah filtered individual XRD images and gave 23 563 indexed shots for (c) and (d).

**FIG. 7.**
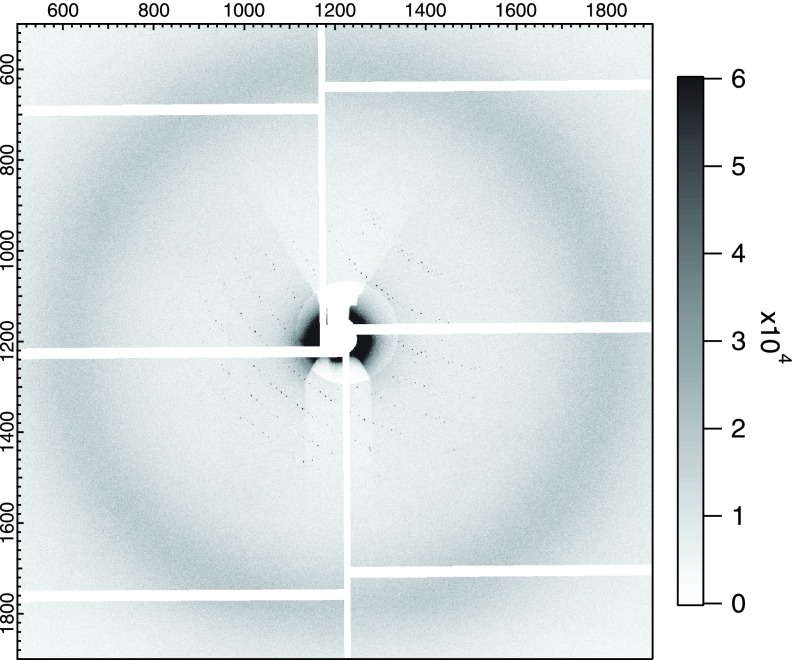
XRD image of PSII microcrystals. The camera distance was set at ∼60 mm.

Figures 6(a) and 6(b) show the Fe Kα and Kβ spectra of the [Fe^II^(phen)_3_]^2+^ solution, respectively. The inset of Fig. 6(b) corresponds to the valence-to-core (vtc) region dominated by contributions from highest occupied valence orbitals. The Kα and Kβ spectral features, i.e., the shapes and peak positions, agree with those of other low-spin Fe^II^ complexes reported in the literature.^35–37^ Compared with the [Fe^II^(phen)_3_]^2+^ solution, PSII microcrystals exhibit spectral broadening in both Kα and Kβ lines and a blue shift of the Kβ_1,3_ peak [see Figs. 6(c) and 6(d)]. Although the Kα and Kβ spectra do not directly probe valence electrons, their spectral shape is sensitive to the number of unpaired 3d electrons (the spin moment) reflecting the magnitude of the 3p(2p)-3d intra-atomic exchange interaction, hence providing information about the oxidation state. Previous studies showed that the increase in unpaired 3d electrons leads to the broadening of the Kα line and the blue shift of the Kβ_1,3_ peak.^37,38^ This indicates that the Fe spin moment of PSII is higher than that of [Fe^II^(phen)_3_]^2+^. Indeed, the PSII dimer has three Fe atoms; one is a nonheme ferrous ion (Fe^2+^) and the other two are ferric ions (Fe^3+^) in the heme groups. The ferric ions contain unpaired 3d electrons and therefore, the average Fe spin state of PSII is larger than zero (S > 0). On the other hand, the [Fe^II^(phen)_3_]^2+^ complex has only one Fe^2+^ ion in the low-spin configuration (S = 0).^39^ Figure 7 shows a typical diffraction image of PSII. The Bragg spots are observable only in a low-q region and the diffraction quality is poor. Although this problem was not addressed in this test experiment, the optimized sample preparation including the postcrystallization treatment will drastically improve the achievable resolution in XRD. The combination of XES and XRD (XDS) will be a powerful tool to gain detailed insights into the reaction mechanism when combined with the pump-probe scheme.

## IV. CONCLUSIONS

We have developed the measurement system for ultrafast experiments, SPINETT, at SACLA. It is designed to cover complementary X-ray techniques and successfully applied to simultaneous measurements of XES and XRD. Combined with an optical laser, SPINETT allows one to track the electronic and structural changes of matter with an ångström and femtosecond spatiotemporal resolution. In future, we plan to fabricate different types of analyzer crystals for XES, which will enable the simultaneous measurement of spectra of different elements, such as Cu, Fe, and Mn, using two Von Hamos spectrometers. This capability is particularly important for understanding the electron transfer processes inside polymetallic complexes or proteins.

## APPENDIX: DATA AVAILABILITY

The data used for Figs. 3(b)–3(d), 5(c), 6, and 7 are available from the corresponding author upon request. All raw data are saved in the SACLA storage system or database.^40^ Figures can be extracted from the “run,” an experimental unit to bundle the consecutive shot-to-shot data. Figures 3(b)–3(d) corresponds to 554536 of BL3. Figure 5(c) corresponds to 798110 and 798112 of BL3. Figures 6 and 7 correspond to 52377–52506 of BL2.
